# Comparative Effect of Individual Herbs and Their Combination on Sucrose Tolerance in Normoglycemic Rats and Assessment of Sensory and Nutritional Attributes of Fortified Bread With Polyherbal Formulation

**DOI:** 10.7759/cureus.77806

**Published:** 2025-01-22

**Authors:** Fauzia Bano, Seema Kanojia, Arvind K Srivastava

**Affiliations:** 1 Department of Food and Nutrition, Era University, Lucknow, IND

**Keywords:** diabetes mellitus, fortification, polyherbal formulation, sensory attributes, synergistic effect

## Abstract

Introduction: Type 2 diabetes mellitus (T2DM) is a substantial worldwide health issue characterized by chronic hyperglycemia and secondary health complications. The current investigation intends to assess the in vivo hypoglycemic potential of selected medicinal herbs (*Olea europaea*, *Momordica charantia*, *Enicostemma littorale*, *Gymnema sylvestre*,* Cinnamomum zeylanicum*,* Garcinia indica, Hibiscus rosa-sinensis*, and *Emblica officinalis*) and their combination and evaluate the sensory and nutritional characteristics of newly fortified bread with polyherbal formulation (PHF).

Methodology: Male Wistar albino rats have been allocated into standard and experimental groups, receiving individual herb crude powder and PHF at different doses, i.e., 25, 50, 75, and 100 mg/kg of dose on post-oral sucrose-loaded (5 mg/kg of body weight) rats. The blood glucose levels have been assessed every 0, 30, 60, 90, and 120 minutes. The area under the curve (AUC) was determined. Both sensory and proximate evaluations were performed on the bread.

Result: The results demonstrated a significant (p<0.05) lowering in the blood glucose levels with 75 and 100 mg/kg of dose at different time intervals in rats treated with PHF in contrast to the control group, while the administration of individual herbs does not illustrate any noteworthy changes in the levels of blood glucose except *E. officinalis*. Similarly, bread fortified with 0.1% PHF is highly accepted in sensory attributes and retained its nutritional components.

Conclusion: The combination of herbs showed a synergistic effect, leading to enhanced glucose regulation in an oral sucrose-loaded rat model, and PHF at 75 and 100 mg/kg of dose possess better hypoglycemic activity as compared to individual herbs. The utilization of PHF in dietary products may be recommended as a nutraceutical product for the mitigation of T2DM.

## Introduction

Type 2 diabetes mellitus (T2DM) is a prevalent metabolic ailment that is growing rapidly across the globe. The incidence of T2DM among individuals over the age of 18 has climbed from 4.7% in 1980 to 8.5% in 2014, and the number of persons with diabetes mellitus (DM) has quadrupled in just 34 years, from 108 million in 1980 to 422 million in 2014 [1]. By 2030, DM is expected to be the sixth most common cause of death worldwide [2]. DM has traditionally been related to both macrovascular and microvascular consequences, such as peripheral neuropathy, retinal degeneration, and DM-related kidney dysfunction, as well as coronary cardiac ailments, stroke, and peripheral arterial disease [3].

Conventional antidiabetic drugs, although effective, often come with significant side effects and limitations. This has led to an increasing interest in exploring alternative and complementary therapies, particularly those derived from medicinal plants. Traditional Indian medicine, with its rich repository of botanical resources, offers numerous plants reputed for their hypoglycemic properties.

The two tenets underpinning the Ayurvedic medicine system are the use of a single herb as a drug and the use of multiple herbs as a drug. This crucial conventional therapeutic approach combines a different kind of therapeutic plant to accomplish the additional medicinal effectiveness, known as polypharmacy or polyherbalism [4]. Certain plants may not have enough bioactive phytoconstituents to provide the necessary therapeutic effect. A polyherbal formulation (PHF) is a mixture of medicinal herbs that is intended to have a higher therapeutic impact than using only one herb or medication in a certain ratio.

Selected medicinal herbs, namely, *Olea europaea* (olives), *Momordica charantia* (bitter gourd), *Enicostemma littorale *(chota-chiretta), *Gymnema sylvestre* (gurmar), *Cinnamomum zeylanicum* (cinnamon), *Garcinia indica *(kokum), *Hibiscus rosa-sinensis *(rose mallow), and *Emblica officinalis *(Indian gooseberry), possess potential bioactive compounds that are responsible for their well-known pharmacological activities. *O. europaea* leaves have many phenolic compounds and flavonoids, mainly gallic acid, syringic acid, oleuropein, vanillic acid, rutin, benzoic acid, etc. Olive leaf extract possesses alpha-glucosidase inhibitory activity, thus helpful in reducing blood sugar levels [5]. *M. charantia* has a wide variety of bioactive chemicals with hypoglycemic potentials; the most well-researched of these were momordicin, charantin, and cucurbitane-type triterpene glycosides. Purified from the fruit and seeds of *M. charantia,* polypeptide-p demonstrated potent hypoglycemic effects [6]. Swertiamarin, a major bioactive component found in *E. littorale*, showed antidiabetic properties [7]. *G. sylvestre* contains a bioactive compound, mainly gymnemic acid. Based on existing research, gymnemic acids are useful in treating noncommunicable disorders like DM, heart disease, cancer, and obesity [8]. One of the main compounds isolated from *C. zeylanicum* is cinnamaldehyde. Compared to metformin, which is frequently used in conventional medicine, it appears to lower plasma glucose levels more successfully. The bioactive components of cinnamon oil increase the expression of proteins essential for insulin signaling, glucose transport, and dyslipidemia control [9]. *G. indica* has been investigated for its potential health benefits and is associated with a variety of bioactive substances, such as citric acids, flavonoids, and phenolic acids. Among these, the main bioactive substances are garcinol, hydroxycitric acid, and anthocyanins. Numerous therapeutic applications for a range of illnesses, including cancer, inflammation, DM, obesity, cardiovascular disease, and neurological diseases, have been reported for *G. indica* [10]. According to a phytochemical study, flavonoids, tannins, terpenoids, saponins, and alkaloids are the primary bioactive substances resulting in *H. rosa-sinensis* therapeutic effects. Recent studies and experiments demonstrated that different kinds of extracts from every part of *H. rosa-sinensis* demonstrated a broad range of advantageous effects, including wound healing, abortifacient, anti-inflammatory, anti-cancer, antioxidant, anti-bacterial, and antidiabetic properties [11]. The main bioactive ingredients in *E. officinalis *are hydrolysable tannins, of which β-glucogallin is the most prevalent, which is responsible for its antidiabetic activity [12].

Nutraceutical products, comprising a considerable quantity of medicinal plants and having suitable organoleptic features, could be appropriate to boost product quality and substitute for the shortage of functional and nutritional constituents. Fortification of bread with PHF enhances the therapeutic aspect and may help to manage T2DM. Through this study, we aim to contribute to the expanding collection of evidence supporting the use of medicinal plants in DM management and to identify promising possibilities for further development into effective hypoglycemic agents.

## Materials and methods

Selection of herbs and dose standardization

The selected Indian medicinal plants were claimed to be antidiabetic and were used for the management of metabolic disorders for many years. The selection of plants was based on their established antihyperglycemic properties as well as pharmacological dosages in various rat models based on prior studies [7,11-16].

Procurement of the crude powder of selected herbs

The crude powder of the selected Indian medicinal plants used in the research includes the leaves of *O. europaea, E. littorale, G. sylvestre,* fruits of *M. charantia, G. indica, E. officinalis,* flowers of *H. rosa-sinensis,* and the bark of *C. zeylanicum* (Table 1). The crude powder of the abovementioned plant parts was procured from the authenticated shops in the local market of Lucknow, India.

**Table 1:** Selected plants for the experiment

Herbs	Part used	Pharmacological dose in rats (mg/kg)
Olea europaea	Leaves	16
Momordica charantia	Fruits	60
Enicostemma littorale	Leaves	75
Gymnema sylvestre	Leaves	100
Cinnamomum zeylanicum	Bark	100
Garcinia indica	Fruit	150
Hibiscus rosa-sinensis	Flower	80
Emblica officinalis	Fruit	150

Preparation of PHF by incorporating selected herbs

The incorporation of selected herbs was based on their antidiabetic activities and dose profiles in experimental rat models. Animal dose was converted into human dose by the formula [17] $\text{Human Effective Dose}\left(\frac{mg}{kg}\right)=\text{Animal Dose}\left(\frac{mg}{kg}\right)&times;\frac{\left(\text{Animal Km}\right)}{\left(\text{Human Km}\right)}$ where Km is the correction factor, animal Km (rats) is 6.2, human Km is 37, and ideal body weight (human) is 60 kg.

Procurement of experimental animals

Male Wistar albino rats weighing 160-200 g of body weight, 5-6 weeks of age, obtained from the Indian Veterinary Research Institute, Izatnagar, Bareilly, India, were involved in the experimental procedure.

Experimental design

In this current research, male Wistar strain albino rats weighing 160-200 g have been used. The animals were kept in a standardized environment after acclimatization for one week and segregated into various experimental groups, with every group containing six animals. Standard maintenance diet and faucet water were provided ad libitum to all experimental groups. All animals have been always kept in a room having a controlled temperature of 23-25℃, 12 hours of light-dark period, and 50-60% humidity. Taking care of animals was evaluated according to the guidelines for animal experiments, which received approval from the Institutional Animal Ethics Committee of Era's Lucknow Medical College and Hospital, Lucknow, India (approval number: IAEC/ELMCH/2/21-9; Committee for the Purpose of Control and Supervision of Experiments on Animals (CPCSEA) registration number: 1652/PO/Re/S/12/CPCSEA).

Comparative in vivo assessment of the hypoglycemic activity of individual herbs and PHF

The hypoglycemic activity in normoglycemic rats was evaluated using the oral sucrose tolerance test (OSTT). For OSTT following 12 hours of fasting, 5 g/kg of body weight sucrose load was given orally to all experimental groups, and the test sample was given 30 minutes prior to the sucrose load. Blood glucose levels were measured at 0 (just after giving the test sample), 30, 60, 90, and 120 minutes by glucometer using glucose strips from the tail vein of rats under light anesthetic conditions.

The rats were classified into 11 groups (each group contains six rats) as follows: NC: normal control group, received only vehicle of carboxymethyl cellulose; MET: standard drug group, administered metformin 100 mg/kg of body weight; OE: experimental group, having *O. europaea* leaf powder 16 mg/kg of body weight; MC: experimental group, having *M. charantia* fruit powder 60 mg/kg of body weight; EL: experimental group, having *E. littorale* leaf powder 75 mg/kg of body weight; GS: experimental group, having *G. sylvestre* leaf powder 100 mg/kg of body weight; CZ: experimental group, having *C. zeylanicum *bark powder 100 mg/kg of body weight; GI: experimental group, having *G. indica* fruit powder 150 mg/kg of body weight; HS: experimental group, having *H. rosa-sinensis* flower powder 80 mg/kg of body weight; EO: experimental group, having *E. officinalis* fruit powder 150 mg/kg of body weight; and PHF: experimental group, having PHF 100 mg/kg of body weight.

Hypoglycemic activity of PHF at different doses

The hypoglycemic activity of PHF was estimated by OSTT in normoglycemic rats. All experimental animals were provided orally with 5 g/kg of body weight sucrose load. The crude powder of PHF was administered 30 minutes prior to sucrose load at 25, 50, 75, and 100 mg/kg of body weight. The assessments of blood glucose levels were performed at 0 (just after giving the test PHF), 30, 60, 90, and 120 minutes.

Fortification of bread with PHF and its nutritional and sensory evaluation

Preparation of Bread

Four different variations of whole wheat bread were prepared by incorporating PHF, i.e., T0 (control), T1 (0.1% PHF), T2 (0.2% PHF), and T3 (0.3% PHF). Whole wheat flour, low-fat milk, baking powder, and baking soda were bought from the regional marketplace of Lucknow, India.

Sensory Evaluation of Fortified Bread

Sensory evaluation was conducted by selecting 15 members of the panelists, five trained, five semi-trained, and five untrained, from the Department of Food and Nutrition, Era University, Lucknow, India. Scoring was done by using a 9-point hedonic rating scale, which is used to assess color, texture, flavor, taste, and overall acceptability.

Proximate Composition of Fortified Bread

Nutritional composition (moisture, fat, fiber, protein, and carbohydrate content) was determined by different Association of Official Analytical Chemists (AOAC) 2000 methods accordingly.

Statistical analysis 

The data was expressed using the mean values±SEM. After examining significant differences between groups using a one-way ANOVA, Dunnett's post hoc test was conducted using GraphPad Prism Version 5.0 (Dotmatics, Boston, Massachusetts, United States/Insight Venture Management, LLC, New York, New York, United States). Values of p<0.05 were considered statistically significant.

## Results

Composition of PHF

The percentage of selected herbs added in PHF is given in Table 2. The added amount of individual herbs in PHF was approximated to the extrapolated human effective dose from the animal dose.

**Table 2:** Composition of PHF PHF: polyherbal formulation

Ingredients	Pharmacological dose in rats (mg/kg)	Calculated human effective dose (mg/kg)	% amount of herb added in PHF
Olea europaea	16	2.60	2.50
Momordica charantia	60	10.0	10.0
Enicostemma littorale	75	12.5	12.5
Gymnema sylvestre	100	16.7	12.5
Cinnamomum zeylanicum	100	16.7	10.0
Garcinia indica	150	25.0	20.0
Hibiscus rosa-sinensis	80	13.3	12.5
Emblica officinalis	150	25.0	20.0

In vivo hypoglycemic activity of selected Indian medicinal plants and comparison of their combination (PHF)

A significant variation in the baseline levels of blood glucose was not found within the groups before the administration of the test sample (Table 3).

**Table 3:** Effect of PHF and its constituent herbs on OSTT in normoglycemic rats N=6 for each group. All experimental groups were compared with the normal control group (NC). Each value is presented in mean±SEM (*: p<0.05; ns: p>0.05) NC: normal control group, received only vehicle of carboxymethyl cellulose; MET: standard drug group, administered metformin 100 mg/kg of body weight; OE: experimental group, having *Olea europaea* leaf powder 16 mg/kg of body weight; MC: experimental group, having *Momordica charantia *fruit powder 60 mg/kg of body weight; EL: experimental group, having *Enicostemma littorale *leaf powder 75 mg/kg of body weight; GS: experimental group, having *Gymnema sylvestre *leaf powder 100 mg/kg of body weight; CZ: experimental group, having *Cinnamomum zeylanicum *bark powder 100 mg/kg of body weight; GI: experimental group, having *Garcinia indica *fruit powder 150 mg/kg of body weight; HS: experimental group, having *Hibiscus rosa-sinensis *flower powder 80 mg/kg of body weight; EO: experimental group, having *Emblica officinalis *fruit powder 150 mg/kg of body weight; and PHF: experimental group, having PHF 100 mg/kg of body weight SEM: standard error of the mean; AUC: area under the curve; PHF: polyherbal formulation; OSTT: oral sucrose tolerance test

Groups	Blood glucose (mg/dl)					AUC (0-120 min)
	0 min	30 min	60 min	90 min	120 min	
NC	63.16±4.57	127.50±4.47	107.16±4.66	99.16±4.88	89.83±3.96	12310
MET	69.66±4.82	93.50±2.71	64.16±2.19	59.16±1.70	53.66±1.17	8355*
OE	83.3±3.07	96.83±6.17	88.16±4.00	85.66±4.07	84.16±3.92	10588^ns^
MC	74.80±5.75	103.60±4.36	98.80±2.43	94.00±3.28	84.80±4.16	11286^ns^
EL	77.33±3.62	116.83±6.88	81.16±3.67	77.66±3.98	71.00±5.03	10495^ns^
GS	74.80±5.75	101.40±4.43	89.20±1.06	82.80±1.53	89.20±1.06	10662^ns^
CZ	86.80±3.38	106.00±2.58	85.41±4.53	85.86±3.72	80.84±2.78	10830^ns^
GI	74.33±3.51	125.07±5.61	97.52±5.96	91.62±5.54	86.33±3.43	11815^ns^
HS	80.00±5.14	109.33±3.11	101.83±1.64	92.83±2.02	86.33±1.78	11615^ns^
EO	61.20±11.99	82.50±5.51	70.66±3.61	63.00±2.70	60.83±2.49	8313*
PHF	78.01±2.69	81.83±1.77	69.51±2.37	65.86±2.46	60.28±2.95	8305*

**Figure 1:**
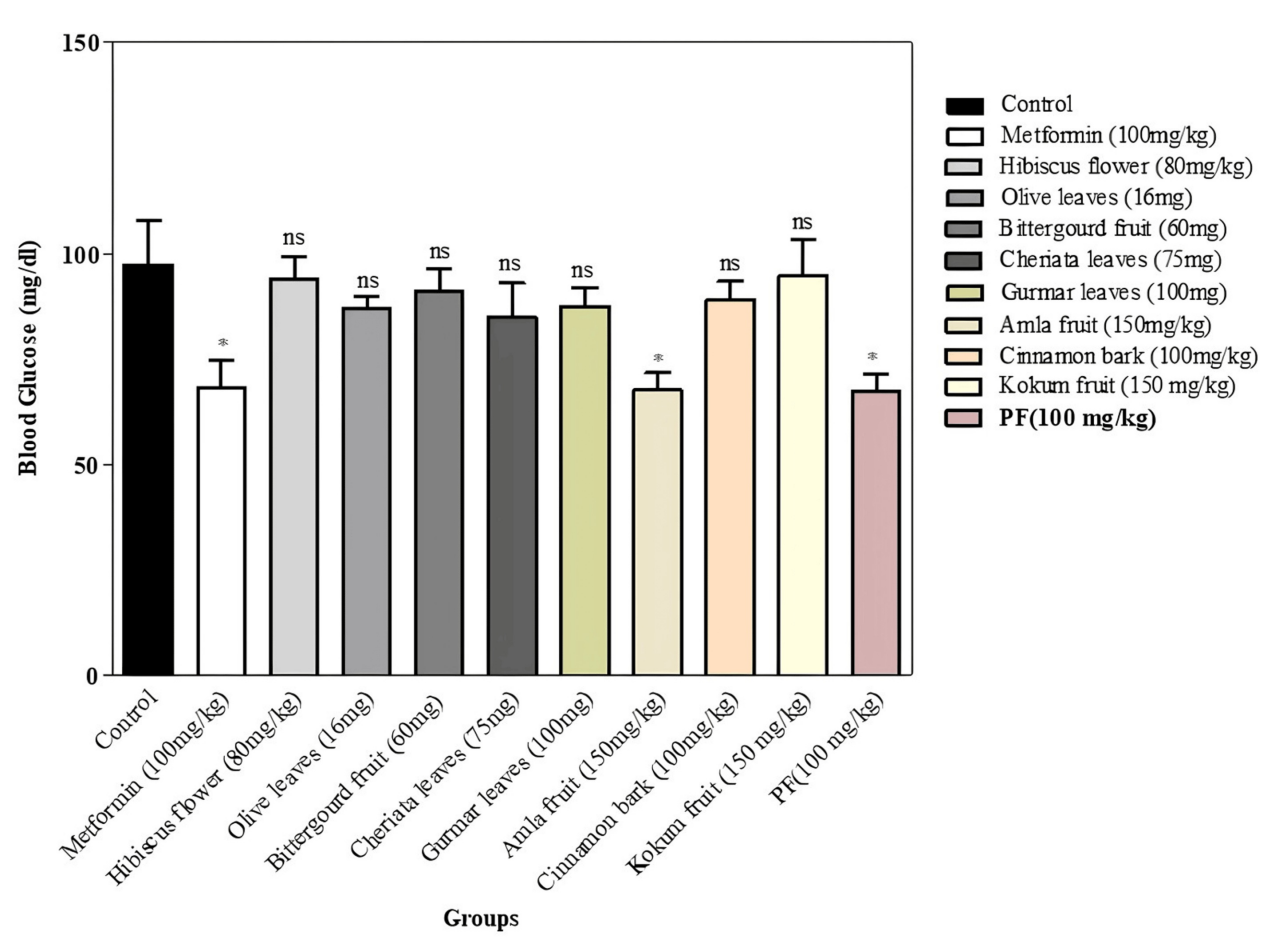
Hypoglycemic activity of different parts of selected herbs and PHF in sucrose-loaded normoglycemic rat model The graph represents the blood glucose levels of the experimental group in comparison with the control group N=6; *: p<0.05; ^ns^: p>0.05 PHF: polyherbal formulation

The measurements of blood glucose were conducted at distinct moments of time, i.e., 0, 30, 60, 90, and 120 minutes, for all experimental and normal control groups. The data was analyzed, and the group with 100 mg/kg of PHF and amla fruit powder showed a remarkable (p<0.05) lowering in the levels of blood sugar as compared to the standard control group (Figure 1 and Table 4). Similarly, a substantial lowering was demonstrated in the group that was supplemented with standard medication, metformin 100 mg/kg, when contrasted with the standard control group. 

**Table 4:** Dunnett's multiple comparison test of blood glucose P<0.05 is considered statistically significant PHF: polyherbal formulation

Dunnett's multiple comparison test	Mean difference	q	95% Cl	P-value	Significance
Control vs metformin (100 mg/kg)	29.33	3.318	4.276 to 54.39	0.047	*
Control vs olive leaves (16 mg/kg)	10.33	1.169	-14.72 to 35.39	0.073	ns
Control vs bitter gourd fruit (60 mg/kg)	6.167	0.6976	-18.89 to 31.22	0.575	ns
Control vs cheriata leaves (75 mg/kg)	12.57	1.422	-12.49 to 37.62	0.422	ns
Control vs gurmar leaves (100 mg/kg)	9.887	1.118	-15.17 to 34.94	0.630	ns
Control vs hibiscus flower (80 mg/kg)	3.300	0.3733	-21.76 to 28.36	0.192	ns
Control vs cinnamon bark (100 mg/kg)	8.407	0.9510	-16.65 to 33.46	0.763	ns
Control vs kokum fruit (150 mg/kg)	2.533	0.2866	-22.52 to 27.59	0.287	ns
Control vs amla fruit (150 mg/kg)	29.77	3.367	4.710 to 54.82	0.0293	*
Control vs PHF (100 mg/kg)	29.90	3.382	4.843 to 54.96	0.0144	*

Hypoglycemic activity of PHF at different doses

**Figure 2:**
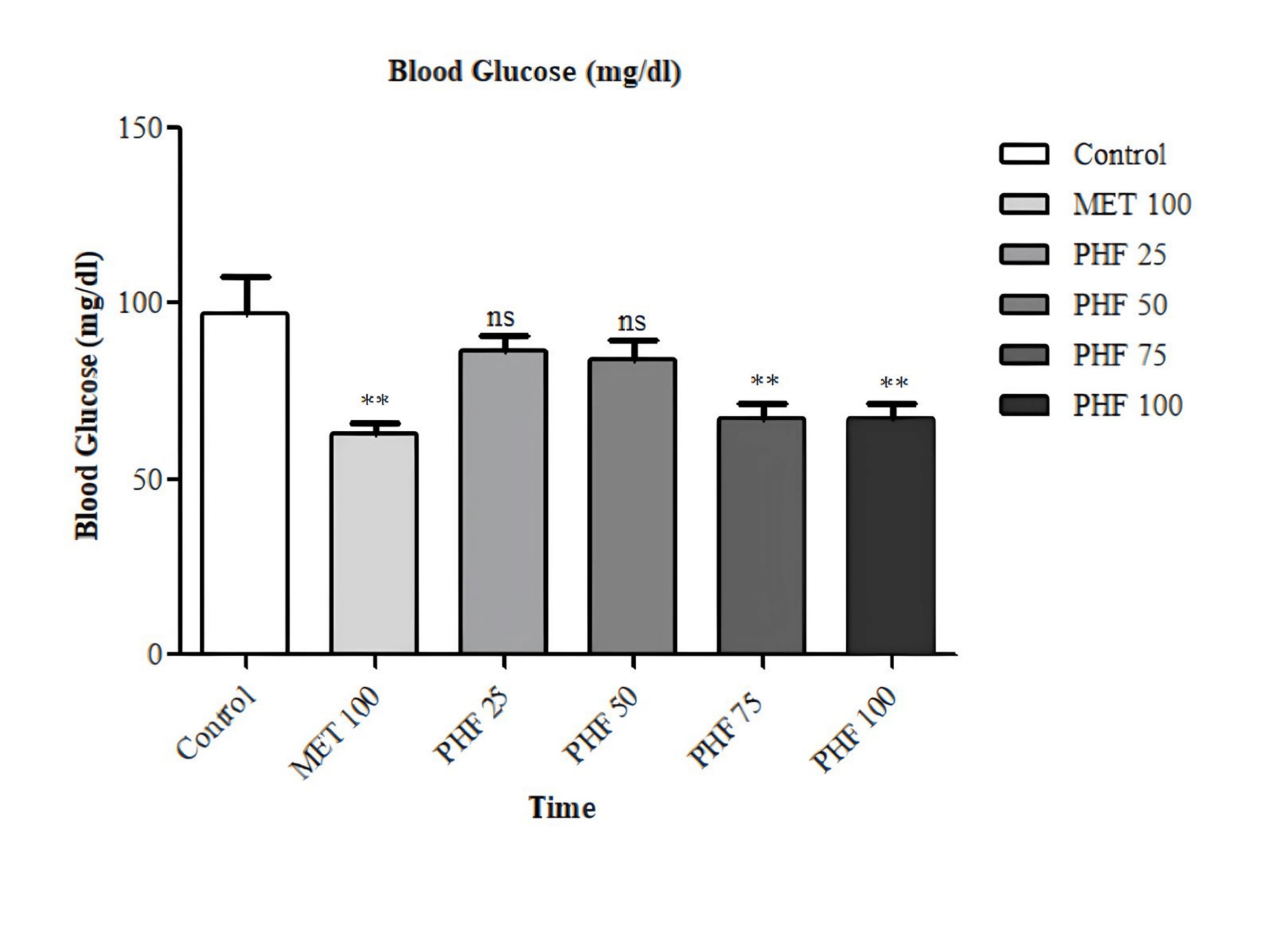
Hypoglycemic activity of PHF at different doses in sucrose-loaded rat model The graph represents the blood glucose levels of the experimental group as compared to the control group N=6; **: p<0.01; ^ns^: p>0.05 PHF: polyherbal formulation

The hypoglycemic activity of PHF was evaluated by an OSTT in normoglycemic rats. In Figure 2, the groups administered with the PHF at doses of 25 and 50 mg/per kg of body weight showed no discernible lowering in the levels of blood glucose, whereas the groups having 75 and 100 mg/kg of PHF showed a remarkable decrease (p<0.01) in the levels of blood glucose as compared to the standard group. Similarly, the group with standard drug, i.e., metformin 100 mg/kg, showed a discernible reduction (p<0.01) in the levels of blood glucose.

Composition of fortified bread

Table 5 presents the data regarding the composition of bread. Four different compositions were formulated with different percentages of PHF. T0 served as a control with 100% wheat flour and 0% PHF, while T1, T2, and T3 served as variations having 0.1%, 0.2%, and 0.3% PHF, respectively.

**Table 5:** Composition of bread PHF: polyherbal formulation

Sample code	Ingredients					
	Whole wheat flour (g)	Instant yeast	Baking powder	Baking soda	Low-fat milk (ml)	PHF (g)
T0 (control)	100.00	1 tsp	1 pinch	1 pinch	10.00	-
T1 (0.1% PHF)	99.90	1 tsp	1 pinch	1 pinch	10.00	0.10
T2 (0.2% PHF)	99.80	1 tsp	1 pinch	1 pinch	10.00	0.20
T3 (0.3% PHF)	99.70	1 tsp	1 pinch	1 pinch	10.00	0.30

Sensory attributes of bread fortified with PHF

Table 6 represents the data of the sensory characteristics of one-way ANOVA.

**Table 6:** Sensory evaluation of fortified bread Each value is presented as mean±SEM. P<0.05 is considered statistically significant SEM: standard error of the mean

Analyzed parameter	Sample code						
	T0	T1	T2	T3	P-value	ANOVA	R^2^
Color	7.84±0.29	8.36±0.23	7.51±1.10	6.97±1.25	0.016*	F=3.79	0.19
Texture	8.23±0.23	8.65±0.20	7.92±0.21	7.15±0.29	0.0005***	F=7.02	0.30
Flavor	7.84±0.29	7.95±0.24	6.68±0.39	5.67±0.41	<0.0001***	F=9.76	0.37
Taste	7.84±0.31	8.11±0.24	6.67±0.42	5.65±0.38	<0.0001***	F=10.53	0.39
Overall acceptability	7.88±0.31	8.22±0.24	7.17±0.39	6.11±0.36	0.0003*	F=7.72	0.32

Color

Between the groups, there are statistically significant variations (p=0.016), and the value of F is 3.79.

Texture

There are statistically substantial variations among the groups in which the F value is 7.02 and the significant value is 0.005.

Flavor

There is substantial statistical variation among all four groups in which the value of F is 9.76 and the significant value is 0.0001. The least significant difference (LSD) in the flavor of the variation was found in the sample having 0.1% PHF as compared to the control sample.

Taste

There is statistically remarkable variation in taste among the groups in which the value of F is 10.5 and the significant value is 0.0001. The LSD was found in the group having 0.1% PHF in comparison to the control group.

Overall Acceptance

There are substantial significant variations found among groups in which the significant value is 0.0003 and the F value is 7.72. There was LSD found in the variation T1, which has 0.1% PHF, as compared to the control one. This indicates that T1 (0.01% PHF) is highly accepted in sensory evaluation as compared to other groups, i.e., T2 and T3, having 0.2% and 0.3% PHF, respectively.

Proximate compositions of fortified bread

The comparative analysis of proximate compositions of fortified bread that are acceptable in sensory attributes with the control group is shown in Table 7.

**Table 7:** Proximate composition of fortified bread Data was presented as mean±SEM, for three repeated measures ns: p>0.05; **: p<0.01 SEM: standard error of the mean

Nutrients (per 100 g)	T0 (before baking)	T0 (control) (after baking)	% nutrient loss in T0	T1 (before baking)	T1 (accepted) (after baking)	% nutrient loss in T1
Total energy (kcal)	381±2.60	354±3.76	7.08^ns^	377±3.74	351±1.89	6.89^ns^
Carbohydrate (g)	76.5±1.81	68.9±2.06	9.85^ns^	73.8±1.67	68.3±2.45	7.49^ns^
Protein (g)	14.9±2.54	13.8±2.68	6.97^ns^	14.5±1.97	13.7±2.57	5.55^ns^
Total fat (g)	1.80±0.76	1.61±1.07	10.5^ns^	1.64±0.82	1.59±2.67	3.04^ns^
Total fiber (g)	11.4±1.79	11.3±1.52	1.04^ns^	11.5±1.84	11.3±1.29	1.65^ns^
Moisture (%)	78.6±2.34	29.9±2.78	61.9**	80.0±4.78	26.2±2.86	67.2**

Considering the outcomes, the addition of 0.1% PHF does not show any remarkable changes in the nutritive content of fortified bead as compared to non-fortified bread.

## Discussion

Although DM is fatal, it can be controlled with the use of some medications, including those from modern and traditional medicine, according to the results of worldwide research conducted so far. Since ancient times, various traditional medical systems have used herbal medicine to treat DM as it is seen to be a safer alternative. Furthermore, compared to a single herb, the treatment using several herbs had superior results and lower toxicity, possibly as a result of the several constituents' synergistic effects [18]. Many of the PHF that have been developed in the last few years have been found to be successful in regulating hyperglycemia in various laboratory models [19].

In this present research, we observed that a PHF including crude powders of *O. europaea*, *M. charantia*, *E. littorale*, *G. sylvestre*, *C. zeylanicum*, *G. indica*, *H. rosa-sinensis*, and *E. officinalis* effectively lowered the blood glucose levels in sucrose-loaded normoglycemic rats in comparison with individual herbs included in the study. The antihyperglycemic effect of selected individual herbs was accomplished in this study. The crude powder of *O. europaea *leaves was administered at 16 mg/kg of body weight in oral sucrose-loaded rats, and its administration did not show any significant lowering of blood glucose levels as compared to the normal control group, while the study conducted by Guex et al. revealed that the long-term treatment of olive leaves was able to reduce glucose levels [20]. Four-week administration of olive leaf extract at 8 and 16 mg/kg doses showed a significant reduction in serum glucose levels in alloxan-induced diabetic rats [13]. Similarly, the administration of leaf powder of *E. littorale *and *G. sylvestre *in sucrose-loaded rat models at 75 and 100 mg/kg, respectively, did not represent any significant change in blood glucose levels monitored till 120 minutes at a 30-minute interval. However, many studies suggested that the administration of *E. littorale *and *G. sylvestre *leaf extract possessed antidiabetic potential [7,8].

The administration of fruit powder of *M. charantia *and *G. indica *at 60 and 100 mg/kg of body weight, respectively, to sucrose-loaded hyperglycemic rats in this research didn't possess any hypoglycemic activity as there was no significant reduction observed in blood glucose levels in OSTT. However, the study conducted by Kolawole et al. showed a significant reduction in blood glucose levels due to the administration of *M. charantia *in alloxan-induced diabetic rats and in normal rats [21]. Similarly, the administration of an aqueous extract of *G. indica *restores erythrocyte glutathione in T2DM rats [22].

The present study revealed that the administration of *E. officinalis *at 150 mg/kg to sucrose-loaded normoglycemic rats showed a significant (p<0.05) reduction in blood glucose levels. Similarly, a study conducted by Majeed et al. revealed that the administration of an extract of *E. officinalis *showed antidiabetic activity [23]. The administration of crude powder of the flower of *H. rosa-sinensis *at 80 mg/kg of dose in oral sucrose-loaded rats did not possess any significant lowering in blood glucose levels, while the study conducted by Pillai and Mini in 2016 found that the administration of a 25 mg/kg dose of petal extract showed significant reduction in the levels of serum glucose (398.56±35.78) [24]. The study conducted by Wariyapperuma et al. showed that cinnamaldehyde, one of the bioactive chemicals in cinnamon, has the ability to block enzymes like α-glucosidase, which is involved in the digestion of carbohydrates. This inhibitor slows down the intestinal absorption of glucose, which can help regulate hyperglycemia [25]. However, in the present study, there was no discernible lowering in the levels of blood glucose reported in cinnamon bark powder administered at 100 mg/kg to the oral sucrose-loaded rat model.

This current investigation demonstrated that the administration of PHF to sucrose-loaded normoglycemic rats resulted in substantial (p<0.05) lowering in levels of blood glucose at 75 and 100 mg/kg of doses, while lower doses at 25 and 50 mg/kg of PHF did not possess any reduction in blood glucose levels. Similar findings were reported in the research by Haye et al., which indicated that the stimulation of the hepatic IRS-PI3K-Akt-GLUT2 signaling pathway, which is mediated by AMPK, helps reduce hyperglycemia, insulin resistance, dyslipidemia, and hepatic architectural degeneration. The PHF reduced the activities of α-glucosidase (IC50 103.0±0.81 μg/mL) and α-amylase (IC50 152.2±9.3 μg/mL), according to in vitro data [26].

Given that a wide range of people eat bread, adding selected medicinal herbs in the form of PHF to bread provides additional health benefits. Taking into account the current study's findings, the fortification of bread with PHF does not affect its sensory attributes, retains its proximate composition, and may also have medicinal qualities to mitigate T2DM. 

## Conclusions

In the current investigation, from the finding, it can be concluded that PHF, i.e., combination of different parts of *O. europaea, M. charantia, E. littorale, G. sylvestre, C. zeylanicum, G. indica, H. rosa-sinensis,* and *E. officinalis*, uphold the hypoglycemic activity, at 75 mg/kg and 100 mg/kg of body weight against oral sucrose-loaded rat models. According to the findings generated that claimed the hypoglycemic activity of PHF, it may be considered a new traditional therapeutic agent for the mitigation of T2DM. Fortification of bread with PHF may be utilized as a nutraceutical food product to combat T2DM. 
